# The protective role of Astaxanthin against oxidative stress and inflammation in peritoneal dialysis rats

**DOI:** 10.37796/2211-8039.1651

**Published:** 2025-06-01

**Authors:** Ratih Tri Kusuma Dewi, Bambang Purwanto, Brian Wasita, Vitri Widyaningsih

**Affiliations:** a Doctoral Program of Medical Sciences, Faculty of Medicine, Sebelas Maret University, Surakarta, 57126, Indonesia; b Department of Internal Medicine, Dr. Moewardi Hospital, Faculty of Medicine Sebelas Maret University, Surakarta, 57126, Indonesia; c Department of Pathological Anatomy, Dr. Moewardi Hospital, Faculty of Medicine Sebelas Maret University, Surakarta, 57126, Indonesia; d Department of Public Health, Faculty of Medicine Sebelas Maret University, Surakarta, 57126 , Indonesia

**Keywords:** Astaxanthin, Peritoneal dialysis, Peritoneal fibrosis, Proinflammatory cytokine, Chronic kidney disease

## Abstract

**Background:**

Prolonged exposure of peritoneal membrane to dialysate solution is known to cause fibrosis mediated by imbalanced inflammatory response and oxidative stress. In this context, proinflammatory cytokines including TGF-β and IL-1β, along with oxidative stress markers namely SOD and MDA, present in circulation, could serve as indicators of fibrosis. This phenomenon has the potential to interfere with the optimization of Chronic Kidney Disease (CKD) treatment. Other oral antioxidant supplements have been widely studied to prevent peritoneal damage, but investigations regarding Astaxanthin (AST) effect on fibrosis marked with proinflammatory cytokine are still limited.

**Aims:**

This study aimed to investigate the protective role of AST supplementation against fibrosis in peritoneal dialysis by evaluating proinflammatory cytokine levels.

**Results:**

The results showed that AST supplementation reduced the levels of MDA, TGF-β, and IL-1β within the circulation while also improving SOD concentration in peritoneal dialysis-induced rats, as indicated by p < 0.001. The longer duration of supplementation, namely 14 vs. 21 days, showed a better outcome against oxidative stress and inflammatory response, as indicated by p < 0.05.

**Conclusion:**

This study found that AST supplementation might prevent peritoneal fibrosis by decreasing MDA, TGF-β, and IL-1β, as well as increasing SOD levels in serum.

## 1. Introduction

Chronic Kidney Disease (CKD) is predicted to become the fifth most common non-communicable disease in 2040, representing a significant global health issue due to the increasing prevalence each year [1]. This disease is associated with an increased risk of morbidity and mortality when not properly treated. The Kidney Disease Improving Global Outcomes (KDIGO) defines CKD as decreased kidney function shown by glomerular filtration rate (GFR) of less than 60 mL/min per 1.73 m^2^ or marked abnormality of structure persisting for more than 3 months [2]. The recommended treatment for patients is renal replacement therapy (RRT), namely hemodialysis and peritoneal dialysis, as well as renal replacement. However, peritoneal fibrosis may arise as a most important complication in patients receiving peritoneal dialysis [3].

Patients undergoing for peritoneal dialysis comprise around 11 % of the total dialysis population [4]. Several studies have shown that peritoneal dialysis excels more than hemodialysis in flexibility, continuity, unit cost, and less invasiveness. However, some risks are associated, such as infectious or non-infectious complications. One of the long-term complications of peritoneal dialysis treatment and ultrafiltration failure is peritoneal fibrosis, initiated by an imbalanced inflammatory response and increased oxidative stress in peritoneal tissue [5]. The explanation of these structural and functional alterations remains unclear, but continuous exposure to bio-incompatible dialysate during peritoneal dialysis is believed to play a crucial role.

High glucose in peritoneal dialysis solution induces peritoneum mesothelial cells (PMCs) to release pro-inflammatory cytokines and chemo-attractants [5]. Inflammatory proteins such as transforming growth factor-beta (TGF-β), tumor necrosis factor-alfa (TNF-α), various interleukins (ILs), and vascular endothelial growth factors (VEGF) are extensively expressed by PMCs and immune cells [6]. Glucose degradation product (GDP) also enhances reactive oxygen species (ROS) in PMCs, mediating peritoneal membrane functional remodeling. In addition, intracellular ROS activates other pro-inflammatory cascades. These events eventually lead to mesothelial cell transition, angiopathy, and membrane fibrosis [5,6].

Current strategies to reduce peritoneal fibrosis include pharmaceutical usage of unfractionated and low-molecular-weight heparins, as well as sulodexide which showed a different response during clinical trials due to its varying capacity to bind complements. According to a previous study, the usage of alanyl-glutamine (Ala-Gln) and molecular hydrogen (H2) produced antioxidant and antiinflammatory effects in various animal models. Future clinical studies are needed to evaluate the efficacy and safety of this therapeutic solution [7].

Exogeneous antioxidant supplement usage in peritoneal dialysis patients has been widely studied. For example, vitamins B, C, D, and E, coenzyme Q10, l-carnitine, a-lipoic acid, curcumin, green tea, flavonoids, polyphenols, omega-3 polyunsaturated fatty acids, statins, trace elements, and Nacetylcysteine were shown to protect patients from peritoneal fibrosis. However, current guidelines for peritoneal dialysis has not offer recommendations regarding the use of exogenous oral supplementation [8].

Astaxanthin (AST) is a natural carotenoid usually found in *Haematococcus pluvialis* algae and several marine organisms. The potent antioxidant and antiinflammatory properties play a crucial role in reducing fibrosis within human organs [9]. According to previous studies, AST has antioxidant effect 100 times stronger than GSH and 1000 times more potent than Vitamin E, an important mitochondrial antioxidative molecule. It also has excellent free radical scavenging properties and renal protective effect through antioxidant mechanism [10]. A study reported that AST showed a promising anti-fibrosis effect by inhibiting ROS activation and suppressing cytokine release. The level of TGF-β1 was increased by renal fibrosis and decreased through AST supplementation. The administration also upregulates the expression of Nrf2, superoxide dismutase (SOD), Interferon type II (IFN-γ), and CD8+ T cells that play a significant role in reducing fibrosis. Additionally, AST restores vascular integrity by inducing vascular endothelial cell growth factor A (VEGF-A) and prevents cell trans-differentiation [11].

AST-containing food supplements are widely used in the health industry and society. Commercial AST is mostly prepared from *H. pluvialis* cultivation, but the synthetic type also plays an important role in preventing the cultivation of algae from its natural habitat and has a positive effect on ecological issues. Both natural and synthetic AST reportedly have a high degree of safety [12], with no acute or chronic genotoxicity or carcinogenicity observed in previous studies [11].

Despite the safety and strong antioxidant property to reduce fibrosis in peritoneal dialysis patients, studies regarding the effect of AST on inflammatory markers such as MDA, TGF-β, IL-1β, and SOD remain limited. Therefore, this study aimed to investigate the effect and potential mechanism of AST supplementation in inhibiting and preventing fibrosis in peritoneal dialysis rats.

## 2. Materials and methods

### 2.1. Ethical statement

This study was conducted in accordance with the Declaration of Helsinki and received approval from the Health Research Ethics Committee of Sebelas Maret University, Surakarta (No. 1.277/XII/HREC/2019). The animals were treated according to the National Institutes of Health Guide for the Care and Use of Laboratory Animals guideline.

### 2.2. Preparation of Astaxanthin (AST)

This study used natural AST manufactured and purchased from SOHO Global Health (Jakarta, Indonesia) in a dosage of 12 mg. This dosage was converted based on the body surface area (BSA) of rats, yielding a dose of 0.216 mg, which was orally administrated once daily.

### 2.3. Animals and experimental design

The experimental procedure was conducted at the Faculty of Medicine, Gadjah Mada University, and the rats used were obtained from the Faculty of Veterinary Medicine. A total of 32 *Sprague Dawley* rats 3–4 months old and 200–300 g body weight were divided randomly into four groups. Rats in all groups were housed in standard cages under the temperature of 23 °C and a 12-h light/dark cycle with free access to laboratory meal and water (ad libitum) adjusted to the body weight. The unilateral ureteral obstruction (UUO) model procedure modeled all subjects for CKD. This procedure induced rats with anesthesia, then the abdomen was incised, and one side of the ureter was ligated with silk 3.0 at two locations.

**Fig. 1:**
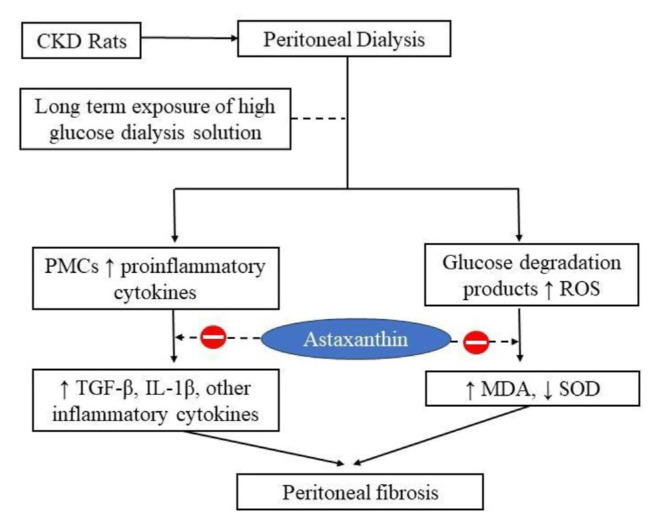
Graphical abstract.

After the induction of the UUO, all groups were subjected to peritoneal dialysis. The subjects were anesthetized and incised 3 cm lengths vertically at the inferior midline of the xiphoid process, then the peritoneal membrane was incised until the cavity was exposed. Afterward, a peritoneal catheter was inserted and fixated with a suture, followed by the injection of 10 ml 4.25 % dialysate solution which flowed up to the cavity (see Fig. 1).

**Fig. 2:**
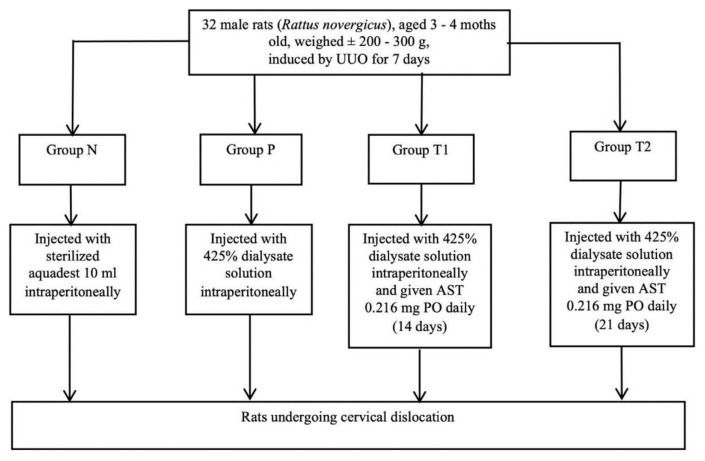
Animal and experimental design.

The first group, the negative control (N), was given only sterilized aquadest injection intraperitoneally (IP), while the other three were administered 10 ml of 4.25 % dialysate solution into the peritoneal cavity daily. The second group, the positive control (P), was administered 4.25 % dialysate solution IP only. Treatment group 1 (T1) was given 4.25 % dialysate solution and 0.216 mg AST orally per day for 14 days, while treatment group 2 (T2) was supplemented with 0.216 mg AST daily for 21 days (Fig. 2). After treatment completion, blood samples were collected, and the rats were subjected to a cervical dislocation procedure.

### 2.4. Biomolecular examination

**Fig. 3:**
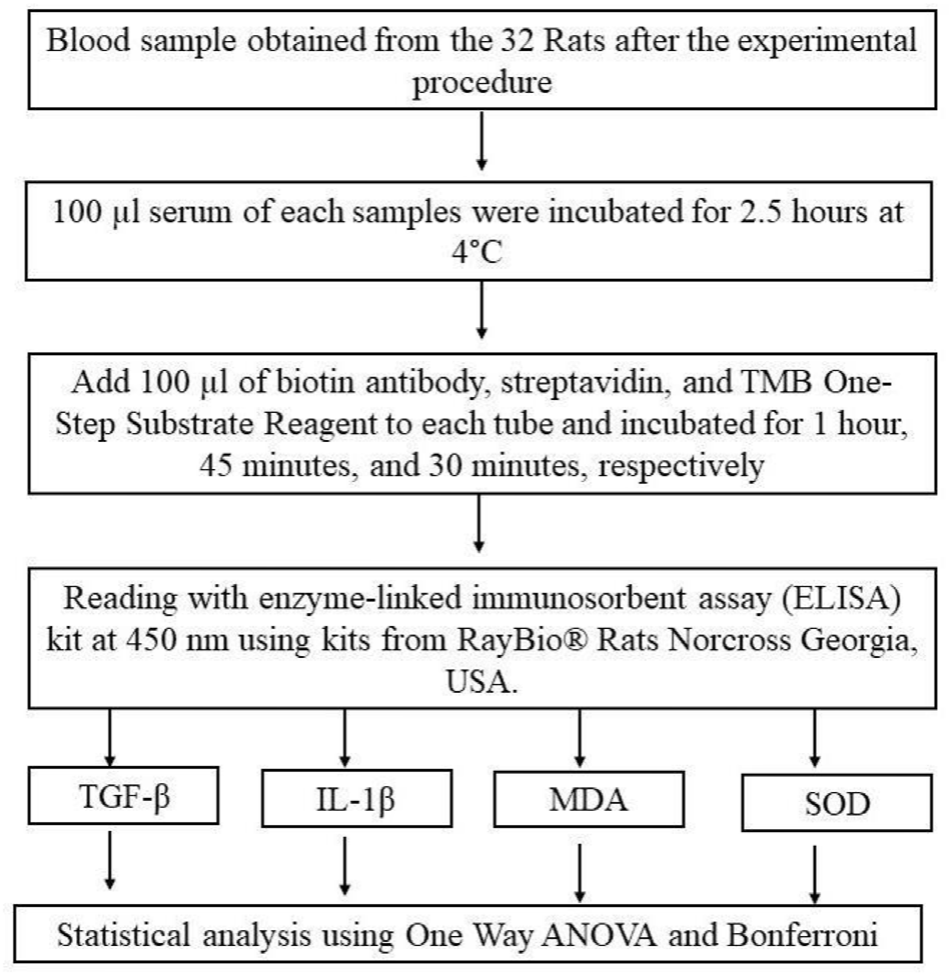
Obtaining blood samples and statistical analysis.

Inflammation and oxidative stress markers were evaluated by examining TGF-β, IL-1β, MDA, and superoxide dismutase (SOD) levels in serum. All markers were measured with enzyme-linked immunosorbent assay (ELISA) using commercial kits according to the manufacturer's instructions. After the reagent and sample were prepared, 100 μl of serum was added into the sterile test tube and incubated for 2.5 h at 4 °C. About 100 μl of each primary antibody was subsequently added to each tube and incubated for 1 h, 45 min, and 30 min, respectively. Reading was performed at 450 nm, and the ELISA Kits used included RayBio® Rats TNF alpha and RayBio® Rats Caspase-3 ELISA Kit, as well as RayBio® Rats MDA, Norcross Georgia, USA (Fig. 3).

### 2.5. Statistical analysis

The collected data were presented as mean ± standard deviation (SD) based on minimum and maximum values. The One-Way ANOVA analysis was conducted to ensure that data was normally distributed. Furthermore, the significant data and intergroup comparisons were analyzed by Bonferroni post hoc multiple comparisons. All analysis was carried out using the Statistical Package for the Social Sciences (SPSS) for Windows version 25.0 (SPSS Inc., Chicago, USA). A p < 0.05 was considered to indicate a statistically significant difference.

## 3. Results and discussion

### 3.1. Effect of AST on MDA and SOD levels

Various markers can be measured to indicate oxidative stress activity, specifically, this study selected MDA level in circulation as an oxidative marker of reactive compound stress. The result showed that AST significantly decreased MDA level after 14 days of supplementation, as indicated by p < 0.001 (Table 1).

Another measurable stress oxidative marker is SOD, an enzyme that plays an antioxidant role and prevents tissue damage. In this study, the group with AST supplementation showed increased SOD levels compared to the control, as indicated by p < 0.05 (Table 2). This implied that AST improved antioxidant activities, thereby obviating further development of peritoneal tissue fibrosis.

In this study, two oxidative stress markers were evaluated, namely MDA and SOD levels. These markers represent oxidative stress signaling pathways regulators and ROS presentation in cells. MDA is a compound produced by lipid peroxidase and an apoptosis promoter [13]. On the other hand, SOD is an enzyme that protects cells from potential damage. Further analysis was conducted to examine the role of AST on these markers in the development of peritoneal fibrosis.

The results showed that MDA level was elevated in groups N and P, while in treatments where.

AST was supplemented for 14 and 21 days, the level significantly reduced, as indicated by p < 0.05.

Although no reference guideline exists for MDA normal range, most studies show that elevated levels were found in malignancy, chronic, and infectious disease cases. This indicates a hyper-oxidative condition in bodies affected with the aforementioned diseases, including CKD.

Rats administered with AST had higher SOD levels compared to those who did not receive the supplement. The difference in SOD levels between the four groups was statistically significant, as indicated by p < 0.05. This suggests that AST has potency in reducing the expression of ROS related to peritoneal dialysis process in rats. Oxidative stress activity inhibited by the antioxidant properties of the supplement reduced the rate of pathological development within tissues, including peritoneal fibrosis in dialysis [11].

Other experimental studies on the antioxidative effect of AST reported a lowered MDA level in rats exposed to radiation when given AST supplementation once daily for eight weeks [14]. This is associated with the ability to absorb excess energy from radiation exposure and convert the free radicals into inactive as well as stable substances. Some varying mechanisms that support this antioxidant activity include: (1) The structure of AST which can turn off the singlet oxygen capacity, (2) Stabilizing membrane structure, reducing fluidity, and membrane permeability, (3) Enhancing activities of antioxidative enzymes (4) Alleviating oxidative damage in mitochondrial, and (5) Inhibiting lipid peroxidation [15].

Another study also investigated the protective effect of AST against lung fibrosis in rats treated with a dosage of 35 mg/kgBW for 7 days. The result showed a significantly lower MDA level in AST group, indicated by p < 0.05. This suggests that AST hinders lung fibrosis development by reducing cell damage caused by lipid peroxidation. Furthermore, supplementation protects renal fibrosis in nephropathy diabetes through signal activation on nuclear factor-erythroid related factor 2 (Nrf2)/antioxidant response element (ARE). AST also has a protective effect on excess oxidative stress and accumulation of fibronectin in glomerular mesangial, which are exposed to a high concentration of glucose [16].

Dialysate fluid in peritoneal dialysis contains high glucose concentration, which induces inflammation of mesothelium tissue. The mechanism of peritoneal fibrosis as one of peritoneal dialysis complications is due to an imbalance of inflammatory response and tissue damage [5,7]. Therefore, this study indicates that AST may prevent fibrosis progressivity by reducing ROS activity in peritoneal dialysis rats, reducing the risk of complications.

Given that oxidative stress and inflammation contribute to the initial events of cardiovascular diseases, antioxidants modulating redox balance, such as AST, may be considered important regulators of inflammatory responses [17]. The antiinflammatory and antioxidant effects were also confirmed by a randomized clinical trial in healthy adult females who received a placebo or dosage of 2 or 8 mg/day. Compared with placebo, AST significantly lowered the levels of the plasma inflammatory marker Creative protein after 8 weeks of treatment [14,18].

### 3.2. Effect of AST on TGF-β and IL-1β levels

TGF-β is one of the tissue profibrotic cytokines and the main regulator in extracellular matrix synthesis.

The analysis results showed significant differences in TGF-β levels within groups. Specifically, groups 3 (T1) and 4 (T2) had lower mean TGF-β levels compared to the control (p < 0.001). This indicates that AST supplementation increased TGF-β and ameliorated peritoneal fibrosis in peritoneal dialysis (Table 3).

IL-1β is a cytokine expressed by cells undergoing inflammation, and it plays a significant role in mediating collagen and fibronectin in peritoneal fibrosis evolution. In this study, AST decreased IL-1β level in the blood, as indicated by p < 0.001 (Table 4).

The significant results of MDA, SOD, TGF-β, and IL-1β levels were further subjected to Bonferroni multiple comparisons to investigate whether the longer duration of AST supplementation had a superior impact as a protector of peritoneal membrane tissue. The results showed that the administration of AST for 21 days had a better outcome than 14 days, as indicated by p < 0.05.

Supplementation for 21 days decreased MDA, TGF-β, IL-1β, and increased SOD, as indicated by p < 0.05, suggesting that longer administration prevented oxidative damage and ameliorated inflammatory response.

The pathogenesis of peritoneal fibrosis is multi-factorial and complex, at the molecular level, immunity response disturbance is a major cause of fibrosis development in peritoneal mesangial cells subsequently activating an inflammatory response. This study evaluated two inflammatory cytokines, namely GF-β and IL-1β [6,7]. Initially, the local inflammatory response is characterized by the formation of TGF-β expressed by various cells. TGF-β activates an inflammatory cascade underneath the Smad2/3 and non-Smad signaling pathways simultaneously [18].

In this study, circulating TGF-β was measured using the ELISA method and the results showed a higher level of TGF-β within the positive control group. However, the groups given AST showed lower TGF-β levels. The statistical analysis results showed that the treatment significantly depleted TGF-β in peritoneal fibrosis rats model, indicated by p < 0.05. There was no significant difference between 14 and 21 days of AST supplementation.

TGF-β is one of the pro-fibrosis cytokines expressed by numerous cells in cases of an immune response. Dialysate fluid may stimulate the synthesis by activating the protein kinase C (PKC) signal in mesothelial cells. This response also occurs systematically, increasing the level of TGF-β in positive control rats. Furthermore, TGF-β initiates fibrogenesis by inducing various mechanisms, such as epithelial–mesenchymal transition (EMT), fibroblast proliferation, and extracellular matrix deposition [7,8,19].

A previous study mentioned that AST supports the restoration of alveoli structure and alleviates the deposition of collagen matrix in lung fibrosis. It also increases NRF2 expression in alveolar epithelial cells, suppressing TGF-β expression [20]. Other studies have reported the anti-fibrosis effect of AST against liver fibrosis by mitigating TGF-β levels in stellate cells. The supplementation for 8 weeks in liver fibrosis reduced extracellular matrix formation by attenuating the expression of *nuclear factor kappa-B* (NF-κB) and TGF-β. The treatment also stabilized matrix metalloproteinase-2 (MMP-2) and tissue inhibitory matrix metalloproteinase-1 (TIMP-1) expression within inflammatory response [11]. Furthermore, the anti-fibrosis effect was reported in a recent study on rats with renal interstitial fibrosis and given AST supplementation in two different doses, namely 50 mg/kg and 100 mg/kg for 7 or 14 days following unilateral ureteral obstruction (UUO). The supplementation suppressed TGF-β/Smad signaling pathways and other inflammatory cytokines, such as IL-1β, TNF-α, and MCP-1 in rats [21].

The results suggest that AST supplementation has a beneficial effect in preventing the progression of peritoneal fibrosis. This study observed a decrease in TGF-β levels after AST supplementation in peritoneal dialysis rats.

Another pro-inflammatory cytokine measured was IL-1β, which played a significant role in fibrosis pathogenesis. In fibrosis, IL-1β regulates collagen and fibronectin, activating PAI-1 I damaged cells. Furthermore, it forms collagen deposition together with TNF-α and also plays a significant role in tissue neovascularization [18].

Dialysate fluid is reportedly acidic, triggering the release of IL-1β and long-term exposure alleviates IL-1β expression as well as stimulates inflammatory response within the tissue. The alteration of peritoneal mesothelium structure and function increases tissue vasculature persistently, accompanied by the thickening of sub-mesothelial structure [19].

Based on the results, the highest level of IL-1β was found in the positive control group. Serum IL-1β levels decreased in rats given AST supplementation for 14 and 21 days. The statistical analysis results showed a significant difference between the four groups, indicated by p < 0.05, suggesting that the administration of AST degraded circulating IL-1β in peritoneal dialysis rats.

AST has been shown to reduce nitric oxide (NO), TNF-α, and IL-1β by suppressing NF-κB expression in rats induced by lipopolysaccharide. TNF-α and IL-1β then activate p38-MAPK signaling pathways and induce other pro-inflammatory cytokines. According to in vitro studies, AST affects the degradation of IL-6 and IL-1β by inhibiting *nuclear factor E2-related factor 2* (NRF2) and NF-κB.

These studies show that AST has great potential in suppressing inflammatory response and preventing tissue fibrosis progression [11,20]. Similarly, in this study, AST supplementation lowered IL-1β levels in rats exposed to dialysate. The administration also alleviated inflammatory response in peritoneal tissue, potentially preventing the formation of fibrosis on dialysis.

## 4. Conclusion

In conclusion, AST supplementation reduced oxidative stress and inflammatory response in peritoneal dialysis rats. This was demonstrated by increased levels of SOD as well as decreased MDA, TGF-β, and IL-1β within circulation. The results underscored the role of AST as an adjuvant treatment to prevent peritoneal fibrosis in patients undergoing dialysis. Although the detailed mechanisms need to be further investigated, the results suggested that AST could be a potential drug for alleviating renal fibrosis.

## Figures and Tables

**Table 1:** Difference in mean levels of MDA (nmol/mL).

Group	n	Mean ± Standard deviation	P value
N	8	9.24 ± 0.52	
P T1	8	9.99 ± 0.26 3.01 ± 0.18	<0.001
	8		
T2	8	2.6 ± 0.21	

N = control negative; P = control positive; T1 = treatment group 1; T2 = treatment group 2.

**Table 2:** Difference in mean levels of SOD (nmol/mL).

Group	n	Mean ± Standard deviation	P value
N	8	83.00 ± 4.21	
P T1	8	58.59 ± 4.09 60.74 ± 5.10	<0.001
	8		
T2	8	73.28 ± 3.82	

N = control negative; P = control positive; T1 = treatment group 1; T2 = treatment group 2.

**Table 3:** Difference in mean levels of TGF-β (pg/mL).

Group	n	Mean ± Standard deviation	P value
N	8	6.29 ± 0.27	
P T1	8	17.91 ± 1.15	<0.001
	8	7.76 ± 0.17	
T2	8	6.83 ± 0.18	

N = control negative; P = control positive; T1 = treatment group 1; T2 = treatment group 2.

**Table 4:** Difference in mean levels of IL-1β (pg/mL).

Group	n	Mean ± Standard deviation	P value
N	8	65.35 ± 6.26	
P T1	8	180.11 ± 14.56	<0.001
	8	87.65 ± 9.15	
T2	8	70.65 ± 7.07	

N = control negative; P = control positive; T1 = treatment group 1; T2 = treatment group 2.
